# Coinfection by *Aspergillus* and *Mucoraceae* Species in Two Cases of Acute Rhinosinusitis as a Complication of COVID-19

**DOI:** 10.1155/2022/8114388

**Published:** 2022-03-14

**Authors:** Payam Tabarsi, Somayeh Sharifynia, Mihan Pourabdollah Toutkaboni, Zahra Abtahian, Mohammad Rahdar, Arefeh Sadat Mirahmadian, Atousa Hakamifard

**Affiliations:** ^1^Clinical Tuberculosis and Epidemiology Research Center, National Research Institute of Tuberculosis and Lung Diseases (NRITLD), Shahid Beheshti University of Medical Sciences, Tehran, Iran; ^2^Chronic Respiratory Diseases Research Center, National Research Institute of Tuberculosis and Lung Diseases (NRITLD), Shahid Beheshti University of Medical Sciences, Tehran, Iran; ^3^Infectious Diseases and Tropical Medicine Research Center, Shahid Beheshti University of Medical Sciences, Tehran, Iran

## Abstract

Acute invasive fungal rhinosinusitis (AIFR) is a life-threatening infection often found in immunocompromised patients. In the COVID-19 era, reports of AIFR have emerged, with high mortality and morbidity rate. This paper presents two cases of COVID-19 associated AIFR with the combined proven fungal etiology of *Aspergillus flavus* and *Rhizopus arrhizus* in case 1 and *Aspergillus fumigatus* and *Rhizopus arrhizus* in case 2. Both patients received liposomal amphotericin B then posaconazole combined with aggressive surgical debridement of necrotic tissues with a favorable clinical outcome. Mixed etiology AIFR can influence the outcome; hence, further studies are required upon this new threat.

## 1. Introduction

The prevalence of invasive fungal infections (IFIs) has increased and become one of the leading causes of mortality and morbidity, particularly in immunosuppressed patients. Most of these infections are described in patients with diabetes mellitus, hematologic malignancies, and solid organ transplantation [1]. Bacterial coinfection in SARS-CoV-2, including *Streptococcus pneumoniae*, *Klebsiella pneumoniae*, or *Haemophilus influenza,* and fungal coinfection, including *Aspergillus* or *Mucoraceae* species have become a serious concern in the management of patients with COVID-19 [2, 3]. Both of these fungal infections have been well described as complications of COVID-19, especially among critically ill patients in intensive care units [4]. Herein, we report two cases of acute invasive fungal rhinosinusitis (AIFR) with combined *Aspergillus* and *Mucoraceae* species.

## 2. Case Presentation

### 2.1. Case 1

**Figure 1:**
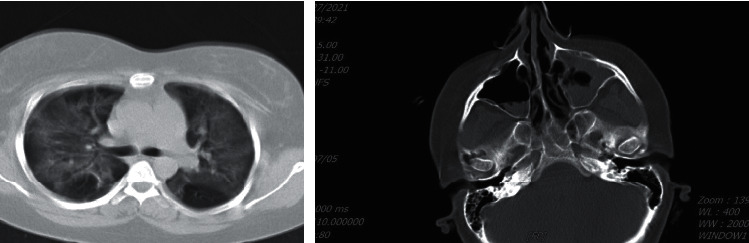
Ostiomeatal complex (OMC) is obstructed leading to mucosal thickening and accumulation of secretions in paranasal sinuses.

**Figure 2:**
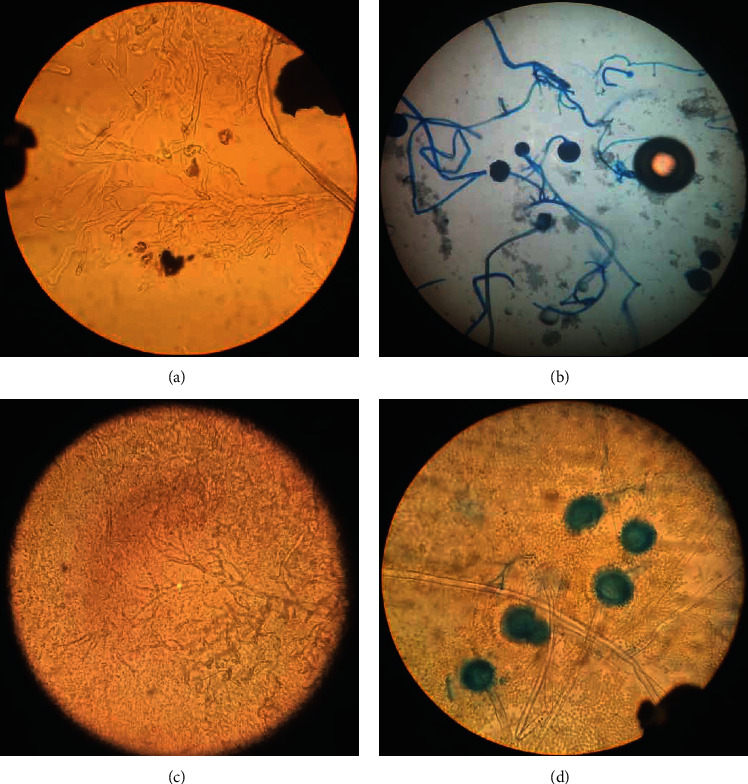
Direct smear revealed hyphae with characteristics consistent with *Mucorales* (a). The result of microscopic examination of cultured fungi was consistent with *Rhizopus arrhizus* (b). Direct smear revealed hyphae with characteristics consistent with *Aspergillus* species (c). The result of microscopic examination of cultured fungi showed *Aspergillus flavus* (d).

**Figure 3:**
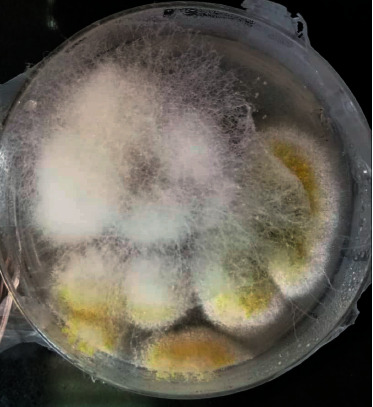
Colonies were identified as *Aspergillus flavus* and *Rhizopus arrhizus*.

The first case was a healthy 31-year-old woman with a five-day history of fever (38.5°C), myalgia, and severe dyspnea (SpO_2_ was 85%–room air), who referred to the emergency department. Reverse transcription-polymerase chain reaction (RT-PCR) confirmed SARS-CoV-2 diagnosis. High-resolution chest CTs obtained at admission showed multifocal areas of consolidation and ground-glass opacification with peripheral and basal predominance. Supportive oxygen therapy with nasal cannula and treatment with remdesivir at a dose of 200 mg on day one and 100 mg/d for five days with methylprednisolone 80 mg every 12 hours were prescribed. Methylprednisolone continued for two days and then switched to dexamethasone 4 mg/d, which continued for additional four days. The patient's condition improved over ten days, and the patient was discharged with SpO_2_ = 93%, afebrile, and improved cough. Several days after discharge, the patient referred to our hospital with a history of headache, left retro-orbital pain, and nasal discharge and was readmitted. At admission, physical examination showed a hemodynamically and respiratory stable patient. The initial laboratory data were as follows: white blood cells (WBC) 13000/*μ*l, platelet (PLT) 240000/*μ*l, hemoglobin (Hb) 10 gr/dl, creatinine (Cr) 0.7, and C-reactive protein (CRP) 26 mg/dl. On physical examination, the extraoral swelling over the left side of the face and the numbness of the left side of the upper lip was remarkable. Subsequently, the computed tomography of sinuses and lungs was performed (Figure 1). According to the mucosal thickening and accumulation of secretions in paranasal sinuses, the patient underwent endoscopic sinus surgery and severe involvement with the necrosis of the left-side lateral nasal wall and left maxillary sinuses were observed. The direct smear of the specimen revealed broad aseptated hyphae and septated hyphae. The sample was cultured with colonies phenotypically identified as *Aspergillus flavus* and *Mucoraceae* species. Sequencing of the ribosomal DNA was performed by amplification, and identification of *Rhizopus arrhizus* (formerly called *Rhizopus oryzae*) was confirmed (Figures 2 and 3). The patient received treatment with liposomal amphotericin B (5 mg kg^−1^ d^−1^). After 15 days of liposomal amphotericin B therapy and removal of necrotic tissues, the therapy switched to posaconazole oral suspension (200 mg every 6 hours). No evidence of recurrence was found at her one-month follow-up.

### 2.2. Case 2

**Figure 4:**
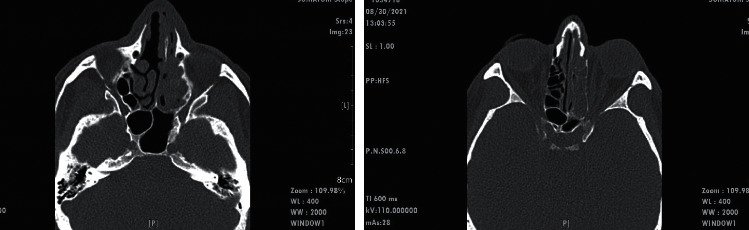
CT scan of sinuses (axial and coronal) revealed nasal septum is deviated to the left side. Opacity is seen in the left maxillary sinus and ethmoidal sinuses.

**Figure 5:**
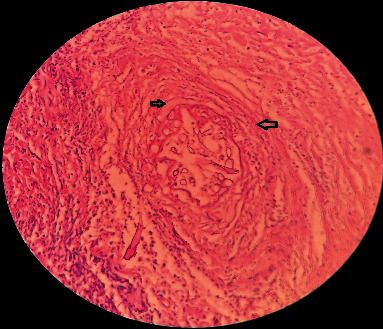
Histopathological examination of tissue sections revealed a vascular structure (large arrow) involved and filled with both thin and broad (smaller arrow) fungal hyphae. (Original magnification ×400).

**Figure 6:**
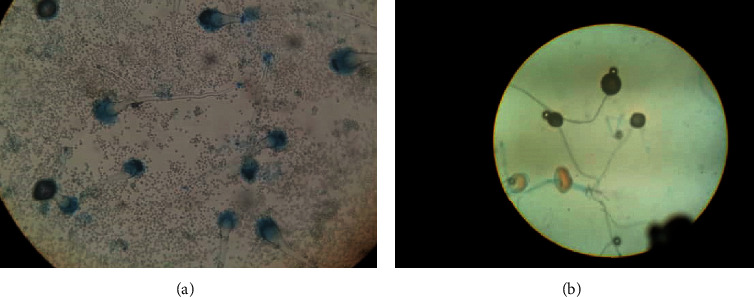
Microscopic examination of cultured fungi revealed results consistent with *Aspergillus fumigatus* (a) and *Rhizopus arrhizus* (b).

**Figure 7:**
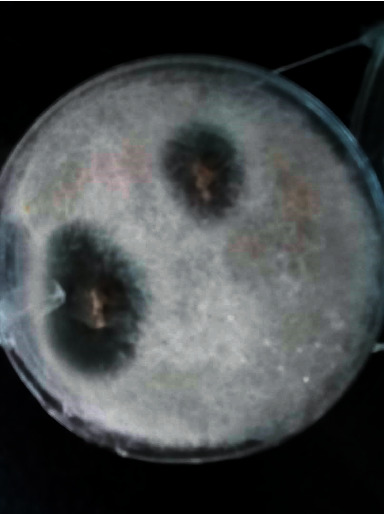
Colonies were identified as *Aspergillus fumigatus* and *Rhizopus arrhizus*.

The second case was a 58-year-old male with a history of diabetes mellitus and hypertension who referred to the emergency department due to nasal obstruction with complaints of purulent discharge, left side facial pain with numbness, and swelling of the upper eyelid and the left cheek. The patient was hospitalized three weeks before the admission with the diagnosis of COVID-19 pneumonia and was treated with remdesivir at a dose of 200 mg on day one followed by 100 mg/d and dexamethasone 8 mg/d for five days. The initial laboratory data were as follows: WBC 7300/*μ*l, PLT 141000/*μ*l, Hb 8.9 gr/dl, Cr 1.4, and CRP 31 mg/dl. SpO_2_ was 92% at room. He had a history of type 2 diabetes mellitus treated with an oral hypoglycemic agent, and his blood sugar level and HbA1c were 210 mg/dl and 6%, respectively. Physical examination showed the ptosis of the left eye associated with periorbital edema and numbness of the left cheek. The findings of the sinuses CT scan were opacity in the left maxillary and ethmoidal sinuses (Figure 4). The endoscopy sinus surgery showed necrotic tissues in the left middle turbinate and left maxillary sinus associated with profuse purulent discharge. The histopathological examination of tissue sections revealed a vascular structure (large arrow) involved and filled with both thin and broad (smaller arrow) fungal hyphae (Figure 5). The sample was cultured with colonies phenotypically identified as *Aspergillus fumigatus* and *Mucoraceae* species. PCR amplification and sequencing was performed, and the *Mucoraceae* species was identified as *Rhizopus arrhizus* (formerly called *Rhizopus oryzae*) (Figures 6 and 7). The patient received treatment with liposomal amphotericin B (5 mg·kg^−1^·d^−1^). After 17 days of liposomal amphotericin B therapy and removal of all necrotic tissues, the therapy switched to posaconazole oral suspension (200 mg every 6 hours). At the time of writing this paper, 14 days follow-up demonstrated no evidence of recurrence.

## 3. Discussion

AIFR is a time-sensitive condition that must be diagnosed and treated promptly to avoid serious complications. The most common etiologic agents of these invasive fungal infections include *Mucoraceae* species and *Aspergillus* species [5]. *Rhizopus oryzae* and *Aspergillus flavus* are the most common organisms isolated from patients with AIFR [6,7].

Mucormycosis is an angioinvasive opportunistic infection caused by fungi belonging to the Mucorales order and the Mucoraceae family. This invasive fungal infection is recognized as one of the most rapidly progressive lethal forms of fungal infection with a high mortality of 70–100% [8,9]. Rhino-orbito-cerebral mucormycosis is the most commonly reported form of the disease characterized by progressive invasion of the hard palate, paranasal sinuses, orbit, and brain [10]. This clinical manifestation of mucormycosis is observed mainly in patients with uncontrolled diabetes or having immunosuppressive conditions, such as corticosteroid treatment with immunosuppressive doses, cancer chemotherapy, hematological stem cell transplants, prolonged neutropenia, and solid organ transplants [11].

*Aspergillus* species are ubiquitous molds found in diverse environments throughout the year. *Aspergillus fumigatus* is the most common and life-threatening fungal pathogen, which is particularly important among immunocompromised hosts. *Aspergillus flavus* more commonly causes rhinosinusitis. Pulmonary aspergillosis and sinusitis are the most common sites of invasive aspergillosis most frequently observed in the setting of immunosuppression status [7, 12–14].

Cases of secondary fungal infections, such as aspergillosis and mucormycosis, have been reported since the emergence of COVID-19, demonstrating the importance of COVID-19-associated invasive fungal infections [15]. Imaging modalities, including CT scan and magnetic resonance imaging, can help to suggest a diagnosis of AIFR, and biopsy is necessary to confirm the diagnosis. Direct mycological and histological examinations are the gold standard for diagnosis. In mucormycosis and aspergillosis, the hyphae are specific for each fungus; *Mucoraceae* presents large, broad aseptated hyphae with right-angle branching, and *Aspergillus* shows septated hyphae that branch at 45° angles [16, 17].

Coinfection with mucormycosis and aspergillosis is a rare entity, and very few cases have been published in the literature. Johnson et al. [18] reported a case of combined probable pulmonary aspergillosis and possible mucormycosis in a diabetic male with COVID-19 in the ICU who was treated with liposomal amphotericin B. Zayet et al. reported a case of cerebro-rhino-orbital mucormycosis and aspergillosis coinfection in a patient with diabetes mellitus with complete recovery after antifungal therapy combined with surgical debridement [19].

Anita et al. reported three cases of mucormycosis combined with aspergillosis caused by *Rhizopus* species and *Aspergillus flavus* with favorable clinical outcomes. The patients were treated with surgical debridement combined with intravenous liposomal amphotericin B, and two cases received step-down therapy with posaconazole. One of the cases was a 52-year-old female with a history of diabetes mellitus [20]. In another report, a 55-year-old diabetic male patient was hospitalized with a three-day history of dyspnea, fever, and generalized malaise. The diagnosis of COVID-19 was confirmed. He was started on methylprednisolone (40 mg twice daily), along with general supportive care and management of diabetes mellitus. The patient presented with ptosis and diplopia on day 10 of discharge and rhino-orbital cerebral mucormycosis caused by mixed proven infections of *Rhizopus arrhizus* and *Aspergillus flavus* were confirmed. The patient received amphotericin B and after 17 days of therapy, the patient recovered clinically. Uncontrolled diabetes mellitus was the main predisposing factor [21].

Several mechanisms in COVID-19 predispose the patients to fungal infections. Endothelial damage and immune dysfunction are known complications of COVID-19, which may provide an opportunity for *Aspergillus* and *Mucoraceae* species to invade tissues. A decline in cell-mediated immunity has been observed, including leukopenia, lymphopenia, and T-cell dysregulation. COVID-19 associated lymphopenia is attributed to the production of cytokines and a decrease in CD4+T and CD8+T cells [22, 23]. In addition, the usage of corticosteroids (CSs) in the treatment of COVID-19 may further predispose the patients to these fungal infections, which are attributed to CS-induced hyperglycemia or immunosuppression status caused by high doses of CS [24,25].

Our data indicate that both patients we presented had AIFR caused by proven aspergillosis and mucormycosis. Patient 1 had no other underlying comorbidity, and COVID-19 infection per se was the main risk factor for predisposing the patient to both infections. However, the CS therapy could possibly play a role in this process. In case 2, the combined risk factors, including COVID-19 infection, diabetes mellitus, CS treatment, and its induced hyperglycemia, contributed to invasive fungal infections.

Timely diagnosis of AIFR is critical to enable early initiation of active antifungal therapy. Complete surgical debridement of necrotic tissues, and when feasible, control of the underlying medical condition are the cornerstones of the therapy. Additionally, empiric antifungal therapy should be based on polyene [11].

This paper presents two cases of COVID-19 associated AIFR with mixed fungal etiology of *Aspergillus flavus* and *Rhizopus arrhizus* in case 1 and *Aspergillus fumigatus* and *Rhizopus arrhizus* in case 2. These two case reports highlight that combined aspergillosis and mucormycosis can present as complications of COVID-19. Mixed etiology AIFR can also influence the outcome; therefore, further studies are required upon this challenging threat.

## Data Availability

No data were used to support this study.
